# Fresh versus frozen embryo transfer for full-term singleton birth: a retrospective cohort study

**DOI:** 10.1186/s13048-018-0432-x

**Published:** 2018-07-16

**Authors:** Junwei Zhang, Mingze Du, Zhe Li, Lulu Wang, Jijun Hu, Bei Zhao, Yingying Feng, Xiaolin Chen, Lijun Sun

**Affiliations:** The Reproduction Center, The Third Affiliated Hospital of Zhengzhou University, 7 Kangfuqian Road, Zhengzhou, 450052 Henan People’s Republic of China

**Keywords:** Frozen embryo transfer, Fresh embryo transfer, Neonatal birthweight, Low birthweight, Small-for-gestational age, Macrosomia, Large-for-gestational age, Congenital malformation

## Abstract

**Background:**

Improvements in vitrification and frozen embryo transfer (FET) technologies have rapidly increased, and some evidence suggests that FET may increase pregnancy rates and lead to more favourable perinatal outcomes. However, the outcome of interest should be offspring safety. Therefore, the primary objective of our study was to investigate whether FET was preferable to fresh embryo transfer (ET) in terms of full-term neonatal birthweight and congenital malformations.

**Methods:**

This was a retrospective cohort study of patients with no pregnancy-related complications who underwent first fresh ETs (*n* = 2059) or FETs (*n* = 2053), resulting in full-term singletons births. Outcome measures were neonatal birthweight, low birthweight (LBW), small-for-gestational age (SGA), large-for-gestational age (LGA), macrosomia and congenital malformations. Additionally, we used logistic regression to adjust for baseline characteristics (age, BMI, No. of embryos transferred and embryo stage) between the two groups.

**Results:**

The mean neonatal birthweight was higher for singletons born after FET than for singletons born after fresh ET (3468.7 ± 475.3 vs. 3386.7 ± 448.1; *p* < 0.001). The frequencies of full-term singleton LBW and SGA after FET were significantly lower than those after fresh ET (1.7% vs. 3.0 and 4.4% vs. 6.7%, respectively), with adjusted rate ratios of 0.59 (95% CI, 0.37 to 0.98; *p* = 0.026) and 0.73 (95% CI, 0.55 to 0.99; *p* = 0.041), respectively. FET resulted in higher frequencies of macrosomia and LGA (15.1% vs 10.2 and 22.8% vs. 17.5%, respectively) than fresh ET, with adjusted rate ratios of 1.43 (95% CI, 1.16 to 1.75; *p* = 0.001) and 1.26 (95% CI, 1.07 to 1.49; *p* = 0.007), respectively. Furthermore, the incidence of congenital malformations was not different between the two groups (1.2% vs. 0.9%), with a rate ratio of 0.288.

**Conclusions:**

After the cycles with pregnancy-related complications were excluded and after adjustments for baseline characteristics, women undergoing FET were associated with a higher neonatal birthweight than women undergoing fresh ET cycles. Additionally, the FET protocol was associated with lower rates of LBW and SGA and higher rates of macrosomia and LGA than the fresh ET protocol. Meanwhile, no difference in the congenital malformation rate was evident between the two groups.

## Background

Over the last decade, the use of frozen embryo transfer (FET) cycles has dramatically risen [1]. According to a report conducted by the Society for Assisted Reproductive Technology (SART), the number of FET cycles has increased by 82.5% between 2006 and 2012, outpacing the increasing rate of fresh embryo transfer (ET) cycles. In the latest European survey, FET cycles accounted for 32.4% of all in vitro fertilization (IVF) and intracytoplasmic sperm injection (ICSI) cycles, which was a significant increase compared with a previous report [2]. The main reasons underlying the increasing trend for FET cycles are threefold. First, a newer vitrification technology has become the dominant method used for embryo cryopreservation, significantly increasing the embryo cryosurvival rate compared with slow-freezing [3]. Second, a rapid rise in single ET, combined with the development of pre-implantation genetic screening (PGS) and pre-implantation genetic diagnosis (PGD), has increased the number of embryos available for freezing [4]. Third, a large number of studies have demonstrated that FET may lead to more favourable perinatal and neonatal outcomes [5–9]. For example, in a large Nordic cohort study, singletons born after FET had a lower risk of PTB, low birthweight (LBW), very PTB, and very PTB plus small-for-gestational age (SGA) than singletons born after fresh ET [8]. According to a recent meta-analysis, singleton pregnancies after FET seem to have better perinatal outcomes than those after fresh ET [10]. Additionally, more favourable neonatal outcomes via FET, including LBW and SGA rates, have been reported [7, 11, 12].

Many of the aforementioned studies have suggested that the neonatal outcomes of FET were more favourable than those of fresh ET; however, these studies were not limited to the first ET cycle, and this limitation is necessary. Furthermore, some observational studies have shown that the rates of maternal complications were different between the two groups [5–7]. Additionally, to the best of our knowledge, maternal pregnancy-related complications, such as gestational hypertension and diabetes, can affect neonatal outcomes, such as neonatal weight, LBW, SGA, and large-for-gestational age (LGA) [13–15]. Thus, whether poor neonatal outcomes, including LBW, SGA, LGA, macrosomia and congenital malformations, are due to pregnancy-related complications or to differences in the type of ET method used is unknown.

Given the limitations of these studies, we aimed to investigate whether an initial FET was preferable to a fresh ET in terms of neonatal outcomes, including birthweight, LBW, SGA, macrosomia, LGA and congenital malformations, for infertile women with singleton full-term births, excluding cycles with pregnancy-related complications.

## Methods

### Population

This was a retrospective cohort study of patients with no pregnancy-related complications who had undergone fresh ETs (*n* = 2059) or FET cycles (*n* = 2053) resulting in full-term (37 weeks ≤ delivery weeks < 42 weeks) singleton births between July 2008 and September 2016 at the Reproductive Center of The Third Affiliated Hospital of Zhengzhou University. Singleton full-term births after first IVF/ICSI cycles were included. We excluded cycles with pregnancy-related complications, including pregnancy-induced hypertension, gestational diabetes mellitus, placenta previa, placental abruption and premature rupture of membranes. Furthermore, cycles with donor oocytes, donor embryos, PGD/PGS, vanishing twins or incomplete records were excluded. For our study, we compared neonatal outcomes, including birthweight, term LBW, SGA, LGA, macrosomia and congenital malformations, of fresh ETs and FETs. To minimize potential bias and confounding, we used logistic regression to adjust for the baseline characteristics and cycle information.

### Control ovarian hyperstimulation and IVF/ICSI

Female patients were subjected to a standardized ovarian stimulation regimen, with protracted down-regulation with gonadotrophin-releasing hormone agonist (GnRH-a) (Diphereline, lpsen, France), followed by daily use of recombinant follicle-stimulating hormone (Gonal-f, Merck Serono, Germany), which was based on the ovarian response. The ovarian response was monitored by three-dimensional ultrasonography and serum hormone levels, and daily doses of Gonal-f were adjusted for ovarian hyperstimulation. Final maturation of oocytes was induced by injecting recombinant Human Chorionic Gonadotropin (HCG, Merck, Darmstadt, Germany) at doses of 5000 to 10,000 IU when at least 60% of follicular diameters were ≥ 18 mm. Vaginal ultrasound-guided oocyte retrieval was performed after thirty-six hours. Routine IVF or ICSI, according to the sperm quality, was performed after oocyte retrieval. Luteal support was started on the day of oocyte retrieval by injecting 60 mg of progesterone (Xianju, Zhejiang, China) or via the intravaginal administration of 90 mg of a progesterone sustained-release vaginal gel (Merck Serono, Germany).

### Embryo transfer

Fresh ET: Fresh ET was performed 3 days after fertilization or with blastocyst transplantation 5 days after fertilization. Based on the recommendations of the American Society of Reproductive Medicine, we transferred up to two 3-day embryos or one 5-day blastocyst. ET was routinely performed under abdominal ultrasound guidance. For cases with severe ovarian hyperstimulation syndrome (OHSS), an endometrial thickness ≤ 7 mm, progesterone levels ≥2 on the HCG trigger day and the presence of uterine fluid, we cancelled fresh ETs, cryopreserved all embryos and subsequently performed FETs to minimize the rate of OHSS and to improve the clinical pregnancy rate.

Frozen ET: Preparation of the endometrium for the FET cycle, according to the specific circumstances of each patient, was necessary and mainly including the following four methods: natural cycles, artificial cycles, down-regulation + artificial cycles and induced ovulation cycles. Natural cycles were applied to patients with regular menstrual cycles and spontaneous ovulation. The vaginal ultrasound examination was performed on the 8th to the 10th day of menstruation. When the diameter of dominant follicles was ≥18 mm, the endometrial thickness was ≥7 mm and urinary luteinizing hormone (LH) was positive, an intramuscular injection of 10,000 IU HCG and FETs was given after 3/5 days. Artificial cycles were suitable for those with polycystic ovary syndrome (PCOS) or those with irregular menstrual cycles, using exogenous oestrogen and progesterone. On the 3rd day of menstruation, vaginal ultrasonography was performed. Oral oestradiol valerate (Progynova, Bayer HealthCare, Germany) was administered 2–3 mg tid, and vaginal ultrasonography was performed 5 days later; the drug dose was adjusted depending on the thickness of the endometrium. In women with endometriosis, adenomyosis or uterine fibroids, before artificial cycles, GnRH-a was used for down-regulation. On the 2nd day of the menstrual cycle, if the endometrium was less than 5 mm, an intramuscular injection of Diphereline was given for down-regulation. After reaching the deregulation standard, the artificial cycle was performed. Ovulation cycles were suitable for patients with hypothalamic pituitary dysfunction or endometrial dysplasia in artificial cycles. Oral clomiphene or letrozole was administered on the 3rd-5th days of the menstrual cycle for a total of 5 days, and when the dominant follicles were ≥ 18 mm and the endometrial thickness was ≥7 mm, HCG was injected to induce ovulation. Follicle and endometrial scanning was performed by vaginal ultrasound, and embryo or blastocyst transplantation was performed using abdominal ultrasound after 3 or 5 days of endometrial development with luteosterone.

### Outcome measures

The primary concern of our study was offspring safety, which was assessed by examining neonatal birthweight, LBW (birthweight < 2500 g), SGA (<10th percentile for gestational age), macrosomia (birthweight ≥4000 g), and LGA (>90th percentile for gestational age) [16]. In addition, we investigated differences in congenital malformations, including Trisomy 13/18/21, congenital heart disease, polydactyly/syndactyly and others, between the fresh ET and FET cycles of singleton full-term children.

### Statistical analysis

All statistical management and analyses were performed using SPSS software, version 22.0.

The one-sample K-S test was used to check for normality (age, BMI, infertile years, gestational weeks and neonatal birthweight). The Wilcoxon rank sum test was used to assess between-group differences in continuous variables with abnormal distributions, and these variables were expressed as the mean ± SD. Categorical variables were represented as the number of cases (n) and the percentage (%). The means from chi-square analyses were used to assess the differences between groups. For neonatal outcomes (LBW, SGA, macrosomia, LGA), logistic regression was used to adjust for the baseline characteristics (age, BMI, No. of embryos transferred and embryo stage) between the two groups. Unadjusted odds ratios and adjusted odds ratios (AORs) with 95% confidence intervals (CIs) were calculated for the above variables. For congenital malformations, chi-square analysis was used to investigate the differences between the two groups. Statistical significance was set at *p* < 0.05.

## Results

### Study population

**Fig. 1:**
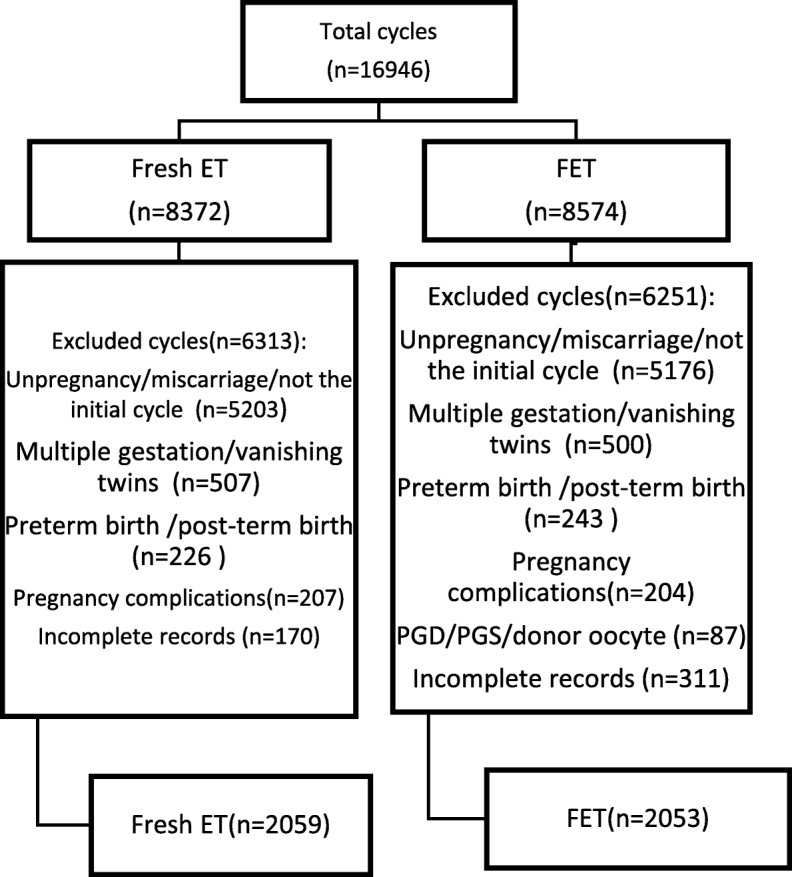
Number of included and excluded cycles

As shown in Fig. 1, 6313 cycles in the fresh ET group and 6215 cycles in the FET group were excluded due to the following reasons: no pregnancy/miscarriage/not the initial cycle (5203 in the fresh ET group and 5176 in the FET group), multiple gestation or vanishing twins (507 in the fresh ET group and 500 in the FET group), preterm birth or post-term birth (226 in the fresh ET group and 243 in the FET group), pregnancy complications (207 in the fresh ET group and 204 in the FET group), PGD/PGS/donor oocyte (87 in the FET group) and incomplete records (170 in the fresh ET group and 311 in the FET group). The remaining 2059 cycles with fresh ETs and 2053 cycles with FETs met the inclusion criteria.

### Characteristics of the study groups

The fundamental maternal characteristics are described in Table 1. Maternal age was higher in the FET group than in the fresh ET group (29.8 ± 4.2 vs. 29.4 ± 4.3; *p* = 0.001). BMI was lower in the fresh ET group than in the FET group (22.4 ± 3.0 vs. 23.3 ± 3.9; *p* = 0.000). More cleavage-stage ETs were included in the fresh ET group than in the fresh ET group, while more blastocyst transfers were included in the FET group than in the fresh ET group (*p* = 0.000). Meanwhile, the rate of caesarean delivery was higher in the FET group (73.5% vs. 85.2%; p = 0.000). Infertile years, the diagnoses of infertility and the number of ETs were comparable between the two groups.

**Table 1:** Material and cycle characteristics between the two groups

	Fresh-ET (*N* = 2059)	FET(*N* = 2053)	Z/χ^2^	*P* value
Age	29.4 ± 4.3	29.8 ± 4.2	−3.230	0.001
BMI	22.4 ± 3.0	23.3 ± 3.9	−6.252	0.000
Infertile years	3.6 ± 2.7	3.6 ± 2.8	−0.135	0.893
Infertile type			0.157	0.692
Primary infertile	1189 (57.7)	1173 (57.1)		
Secondary infertile	870 (42.3)	880 (42.9)		
Diagnosis of Infertile			1.590	0.662
Tube factor	975 (47.4)	982 (47.8)		
Male factor	505 (24.5)	503 (24.5)		
Male+female	351 (17.0)	364 (17.7)		
Other/unknown	228 (11.1)	204 (9.9)		
No. of embryos transfer	1.9 ± 0.4	1.9 ± 0.6	−2.165	0.030
Embryo stage			373.62	0.000
D3	1855 (90.1)	1333 (64.9)		
D5	204 (9.9)	720 (35.1)		
Delivery mode			86.116	0.000
Vaginal	545 (26.5)	303 (14.8)		
Caesarean	1514 (73.5)	1750 (85.2)		
Gestational weeks	39.0 ± 1.0	39.0 ± 1.1	−2.791	0.005
Offspring sex			0.524	0.469
Male	1077 (52.3)	1097 (53.4)		
Female	982 (47.7)	956 (46.6)		

### Neonatal outcomes between fresh embryo transfer and FET

As shown in Table 2, the mean neonatal birthweight of singletons born after FET was higher than that of singletons born after fresh ET (3386.7 ± 448.1 vs. 3468.7 ± 475.3; p = 0.000). The frequencies of full-term singleton LBW and SGA after FET were significantly lower than those after fresh ET (1.7% vs. 3 and 4.4% vs. 6.7%, respectively), with adjusted rate ratios of 0.59 (95% CI, 0.37 to 0.98; *p* = 0.026) and 0.73 (95% CI, 0.55 to 0.99; *p* = 0.041). However, the frequency of macrosomia was higher after FET than after fresh ET (15.1% vs. 10.2%), with a rate ratio of 1.43 (95% CI, 1.16 to 1.75; *p* = 0.001). The incidence of LGA was significantly higher in the FET group than in the fresh ET group (22.8% vs. 17.5%), with a rate ratio of 1.26 (95% CI, 1.07 to 1.49; *p* = 0.007).

**Table 2:** Neonatal outcomes in live singleton birth after fresh ET and FET

	Fresh-ET (*N* = 2059)	FET (*N* = 2053)	*P* value	Adjusted OR(95%CI)	Adjusted *P* value
Birthweight	3386.7 ± 448.1	3468.7 ± 475.3	0.000	–	–
LBW	62 (3.0)	35 (1.7)	0.006	0.59 (0.37–0.98)	0.026
Macrosomia	209 (10.2)	310 (15.1)	0.000	1.43 (1.16–1.75)	0.001
SGA	138 (6.7)	91 (4.4)	0.002	0.73 (0.55–0.99)	0.041
LGA	360 (17.5)	469 (22.8)	0.000	1.26 (1.07–1.49)	0.007

LBW: low birthweight;SGA: small-for-gestational age;LGA:large-for-gestational age

Adjusted for age, BMI, No. of embryos transfer and embryo stage

### Congenital malformations

There was no significant difference between the rates of congenital malformations (1.2% vs. 0.9%; *p* = 0.288), including Trisomy 13/18/21 (0.4% vs. 0.3%; *p* = 0.596), congenital heart disease (0.3% vs. 0.15%; *p* = 0.207), polydactyly/syndactyly (0.2% vs. 0.15%; *p* = 0.482) and others (0.2% vs. 0.3%; *p* = 0.759), between the two groups (Table 3).

**Table 3:** Congenial malformation between the groups

	Fresh ET(*N* = 2059)	FET (*N* = 2053)	χ^2^	P
Neonatal malformation	25 (1.2)	18 (0.9)	1.131	0.288
Trisomy 13/18/21	8 (0.4)	6 (0.3)	0.281	0.596
Congenital heart disease	7 (0.3)	3 (0.15)	1.592	0.207
Polydactyly/syndactyly	5 (0.2)	3 (0.15)	0.495	0.482
Others	5 (0.2)	6 (0.3)	0.094	0.759

## Discussion

In summary, in the current single-centre retrospective cohort study of 2059 fresh ET cycles and 2053 FET cycles with full-term singleton births, we found that children born after FET were associated with higher birthweights those born after fresh ET cycles. The rates of LBW and SGA were significantly lower for neonates born after FET cycles than for neonates born after fresh ET cycles. FET was associated with a significantly higher rate of macrosomia and LGA. However, the rate of congenital malformations in singleton live births via FET was comparable to the rate via fresh cycles.

### Comparison with other reports

This study is an important supplement to the existing literature, and these findings confirm that FET in may lead to lower risks of LBW and SGA, a higher neonatal birthweight, and higher rates of macrosomia and LGA than fresh ET. Although this finding was consistent with the conclusions of many clinical studies, experimental studies, systematic reviews and meta-analyses, the literature on patients without pregnancy complications, such as pregnancy-induced hypertension, gestational diabetes mellitus, placenta previa, placental abruption and premature rupture of membranes, was limited. This distinction was necessary because pregnancy complications were associated with neonatal outcomes, including LBW, SGA, macrosomia and LGA. For example, a population-based study showed that preeclampsia increased the risk of VLBW [13]. Similarly, Lynn M. et al. [15] showed that chronic hypertension contributed to a high occurrence of SGA, after adjusting for important confounders. Additionally, neonates born of patients with diabetes had increased odds of macrosomia and LGA [17] [18]. Therefore, excluding patients with pregnancy complications, which would have had a significant effect on the neonatal outcomes, was crucial to investigate the differences between fresh ET and FET.

A higher birthweight, lower rates of LBW and SGA, and higher rates of macrosomia and LGA of singletons born after FET has been reported multiple times [7, 8, 19, 20]. In a follow-up study, singletons born of vitrified blastocysts had significantly higher birthweights than those born after fresh ETs, after adjusting for parity and BMI [11]. F. Belva et al. [19] reported that singletons born after FET (*n* = 960) showed a higher birthweight and lower SGA rate than those born after fresh ET (*n* = 1374). In another study conducted by Antonina Sazonova [7], the rates of LGA and macrosomia were higher for FET cycles than for fresh ET cycles. However, these studies were not restricted to the first transfer cycle. Limiting the study to the first ET cycle was necessary because the first ET may select the best-quality embryo to be transplanted, whereas the quality of the frozen embryo following transplantation may differ from the quality of the fresh ET. Thus, to eliminate this confounding factor, in our study, we included only the first ET cycle.

Regarding congenital malformations, F. Belva et al. [19] suggested that both the total major malformation and minor malformation rates were comparable between the fresh embryo group and vitrified embryo group. In another retrospective cohort study, Shi et al. reported that the rate of combined major and minor congenital malformations was not significantly different between the vitrified cleavage-stage embryo group and fresh cleavage-stage group (1.42% vs. 0.62%) [21]. Furthermore, in a larger single-centre cohort study of 6623 delivered singletons, the vitrification of embryos/blastocysts did not increase the rate of birth defects following single ET [22]. However, some studies have reported that FET resulted in a higher major malformation rate in live births [23]. Our study included both the cleavage-stage embryos and blastocysts of the first transfer cycle that resulted in singleton births for both FETs and fresh ETs and excluded cycles with pregnancy-related complications, which had potential effects on neonatal health. We found that there was no difference in the congenital malformation rate between the two groups.

### Plausible biological mechanisms

Based on previous studies, FET cycles are considered to have several potential advantages over fresh ET in terms of perinatal outcomes and offspring safety. Multiple biological mechanisms have been proposed for the negative impacts of fresh transfers on neonatal safety.

In fresh ET cycles, controlled ovarian hyperstimulation (COH) results in a supraphysiological oestradiol environment, which has been hypothesized to affect embryo implantation [24–26]. Furthermore, COH may negatively affect endometrial receptiveness [24]. Additionally, the improved perinatal outcomes after FET may be associated with the absence of COH, which has adverse effects on the endometrium [27]. Additionally, clinical research has provided relevant evidence. In a recent retrospective cohort study of normal responder patients, N. Pereira et al. have reported that the odds of full-term LBW were 6.1–7.9 times higher with E2 levels of > 2500 pg/ml than with the E2 levels of the reference E2 group (E2 < 500–1500 pg/ml) [28]. Furthermore, several investigators have suggested that the hyperoestrogenic milieu with fresh ET cycles may be related to suboptimal endometrial perfusion. For example, Ernest Hung Yu Ng et al. [29, 30] investigated the differences in subendometrial blood flow between stimulated cycles and natural cycles via three-dimensional power Doppler ultrasound with several ultrasound indexes, and the report suggested that endometrial and subendometrial blood flow was significantly lower in the stimulated cycles than in the natural cycles and that serum E2 levels had negative effects on endometrial blood flow in IVF cycles. Additionally, Lee et al. have shown that fresh ET cycles are improved with advanced endometrial angiogenesis after gonadotrophin stimulation [31]. One report presented a hypothesis that controlled ovarian stimulation (COS) was associated with advanced endometrial maturation, resulting in the asynchrony of the endometrium and embryo, undermining the implantation window [32].

Gonadotrophin treatments in COS cycles, as performed during IVF/ICSI, may negatively affect the expression of genes associated with transcriptional activity, thus affecting embryo implantation, placental development and subsequent foetal growth [1, 24, 25, 33]. In addition, D. Haouzi [24] have proposed that when the receptiveness of the endometrium is seriously compromised by the COS protocol, fresh ET should be cancelled, the embryo should be frozen, and thawed embryo replacement should be performed under natural cycles. Moreover, the processes of freezing (cryopreservation or vitrification) and thawing, which function like filters that remove poor-quality embryos, may result in embryos that do not survive [34].

On the other hand, FET was associated with statistically significantly higher rates of LGA and macrosomia than fresh ET. Some clinical studies have suggested that the risks of LGA and macrosomia for FET were not only increased compared with fresh ET but also increased compared with spontaneous neonatal outcomes [8, 35, 36]. The biological mechanism for the effect is unknown, and most of the relevant studies have been performed using animals. The possible biological mechanism may be as follows. In vitro culture methods and culture media may induce a higher offspring birthweight among cattle and sheep, suggesting a varying ability to affect genomic imprinting between offspring via in vitro culture [37]. After the freezing and thawing of embryos, these effects on genomic imprinting can be maintained or be more pronounced. Another possible explanation might be in the interaction of the cryoprotectants used with the main enzyme involved in epigenetic programming [38]. In our study, we also found that FET was associated with a significantly higher rate of LGA and macrosomia than fresh ET. Regrettably, we do not have an explanation for this.

### Strengths and limitations

The strengths of this study are twofold. First, we included single-centre cycles, cycles performed during the same period, and first transfer cycles, which minimized potential bias (e.g., the protocols for ovarian stimulation, application of drugs, laboratory technology, conditions). We also included a large amount of data on perinatal outcomes, as a complement to previous studies. Second, we excluded cycles with pregnancy-related complications, including pregnancy-induced hypertension, gestational diabetes mellitus, placenta previa, placental abruption and premature rupture of membranes, which are associated with adverse perinatal outcomes, such as LBW and SGA. Previous relevant studies comparing neonatal birthweight between the two groups did not exclude these cycles.

Our study also has several limitations. Our study is limited by its retrospective nature, and thus, a further prospective study is needed to validate and verify that FET is associated with lower LBW and SGA rates, higher birthweight, and higher LGA and macrosomia rates. Another limitation is that we did not explore the relevant biological mechanism.

## Conclusion

Our results indicate that FET is associated with lower rates of LBW and SGA than fresh ET cycles, even after adjusting for confounders. This finding is consistent with those of previous studies. However, FET protocols are associated with a higher neonatal birthweight and higher risks of macrosomia and LGA than fresh ET. Additionally, our study demonstrates that freeze-only protocols do not adversely affect the rate of neonatal malformations, including Trisomy 13/18/21, congenital heart disease, polydactyly/syndactyly and others. Regarding LBW and SGA, FET protocols do not have adverse effect compared with fresh cycles. However, the confirmation of the increased risk of LGA and macrosomia after FET warrant further study. FET has become an important supplement to fresh ET; however, whether FET should be the first choice for ET requires further clinical and sic medical research.

## Abbreviations

FET: Frozen embryo transfer
ICSI: Intracytoplasmic sperm injection
IVF: In vitro fertilization
LBW: Low birth weight
LGA: Large-for-gestational age
SGA: Small-for-gestational age
